# Supplementary material for: Self-Assembling Systems Based on Pillar[5]arenes and Surfactants for Encapsulation of Diagnostic Dye DAPI
Source: Int J Mol Sci. 2021 Jun 3;22(11):6038. doi: 10.3390/ijms22116038 (PMC8199762; doi:10.3390/ijms22116038)

Novel self-assembling systems based on pillar[5]arenes and surfactants for encapsulation of diagnostic dye DAPI

Anastasia Nazarova, Arthur Khannanov, Artur Boldyrev, Luidmila Yakimova*, and Ivan Stoikov*

A.M.Butlerov Chemical Institute, Kazan Federal University, 420008 Kremlevskaya, 18, Kazan, Russian Federation; anas7tasia@gmail.com (A.N.); arthann@gmail.com (A.Kh.); boldyrev25@gmail.com (A.B.); mila.yakimova@mail.ru (L.S.).

* Correspondence: ivan.stoikov@mail.ru; Tel.: +7-843-233-7241
mila.yakimova@mail.ru; Tel.: +7-843-233-72-41

Electronic Supplementary Information (18 pages)

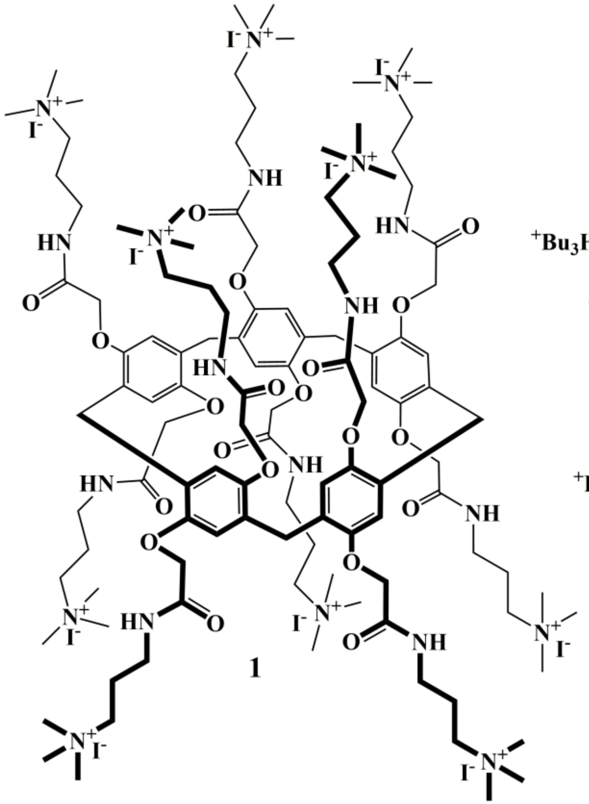

1

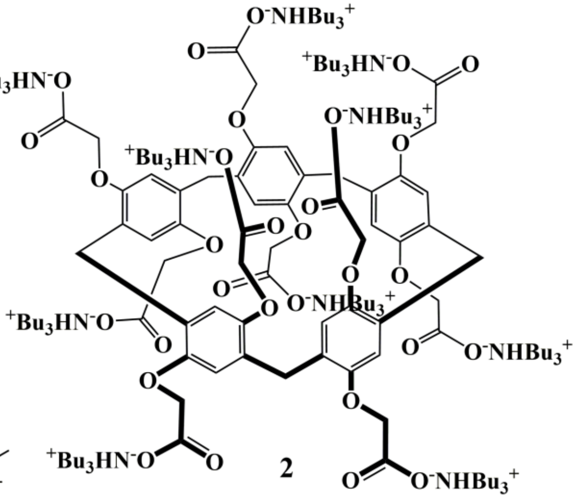

2

Fig. S1. ¹H NMR spectrum of 4,8,14,18,23,26,28,31,32,35-Decakis-[(N-(3′,3′,3′-trimethylammoniumpropyl)carbamoylmethoxy]pillar[5]arene decaiodide (1), DMSO-d_6, 298 K, 400 MHz.

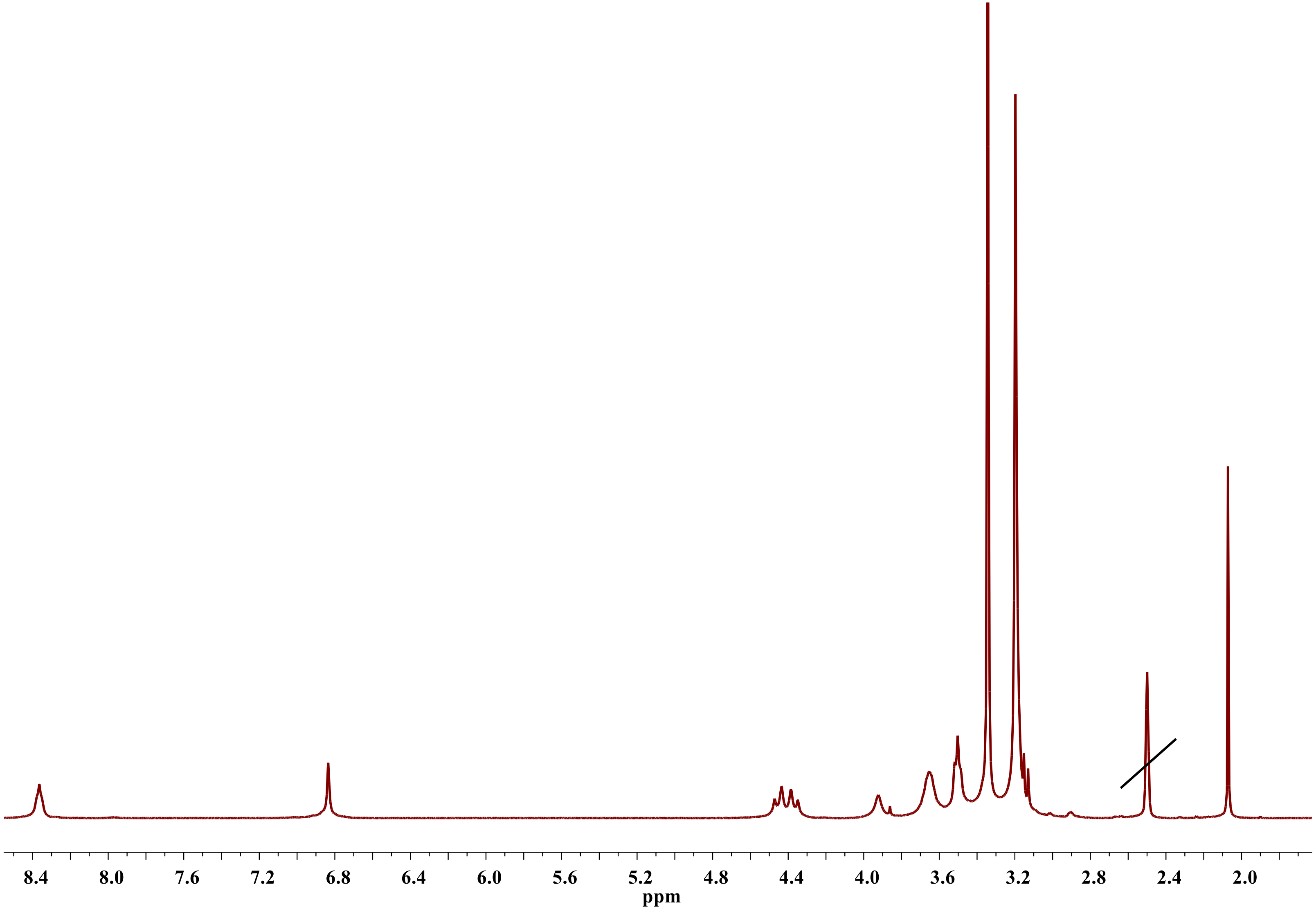

Fig. S2. ^{1}H NMR spectrum of 4,8,14,18,23,26,28,31,32,35-Deca(carboxymethoxy)pillar[5]arene tributylammonium salt (2), D_2O, 298 K, 400 MHz.

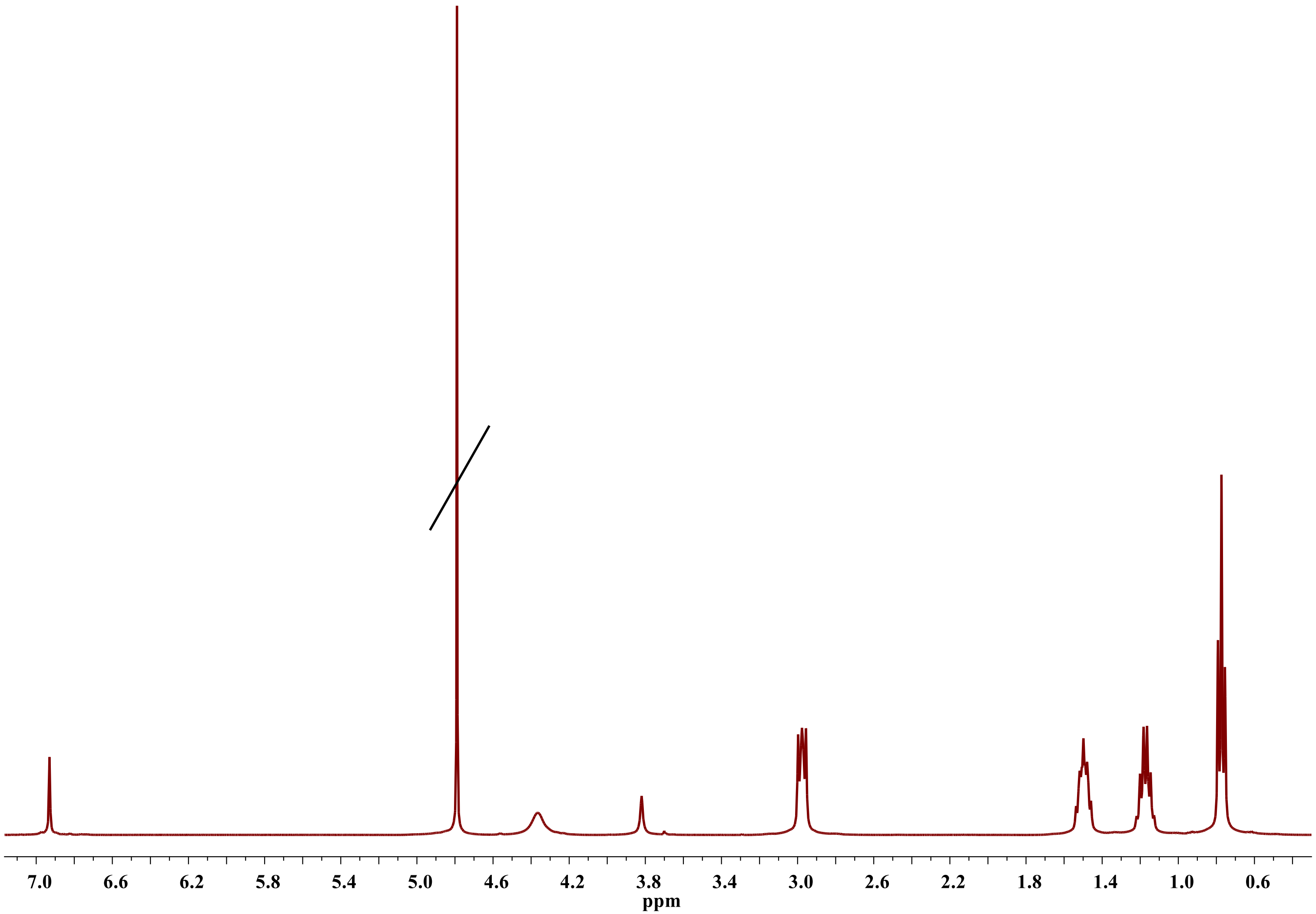

Table S1. Sizes of aggregates (diameter (d) of compounds 1, 2, SDS and DTAC) and the corresponding polydispersity indices (PDI).

Compound	C, M									
	**1×10^{-6}**		**1×10^{-5}**		**1×10^{-4}**		**1×10^{-3}**		**1×10^{-2}**	
	d, nm	PDI	d, nm	PDI	d, nm	PDI	d, nm	PDI	d, nm	PDI
**1**	372±58	0.52	275±36	0.34	347±98	0.44	264±48	0.33	-	-
**2**	583±173	0.86	458±121	0.73	359±75	0.54	160±72	0.57	-	-
**SDS**	171±81	0.74	781±267	0.55	598±135	0.64	252±80	0.49	196±87	0.67
**DTAC**	650±235	0.53	1178±392	0.75	505±168	0.71	475±153	0.58	171±18	0.31

Determination of the stability constant and stoichiometry of the complex by the UV titration

The UV measurements were performed with "Shimadzu UV-3600" instrument. A 3×10^{-3} M solution of **SDS (DTAC)** (30, 60, 90, 120, 150, 180, 210, 240, 270 and 300 μL) in water was added to 0.3 mL of a solution of **1 (2)** (3×10^{-4} M) in water and diluted to final volume of 3 mL with water. The UV spectra of the solutions were then recorded. The stability constant of complex was calculated by Bindfit. Three independent experiments were carried out for each series.

Fig. S3. a) UV-Vis spectra of mixtures of pillar[5]arene 1 (3×10^{-4} M) with different concentrations of SDS in water; b) UV-Vis spectra of mixtures of pillar[5]arene 2 (3×10^{-4} M) with different concentrations of DTAC in water.

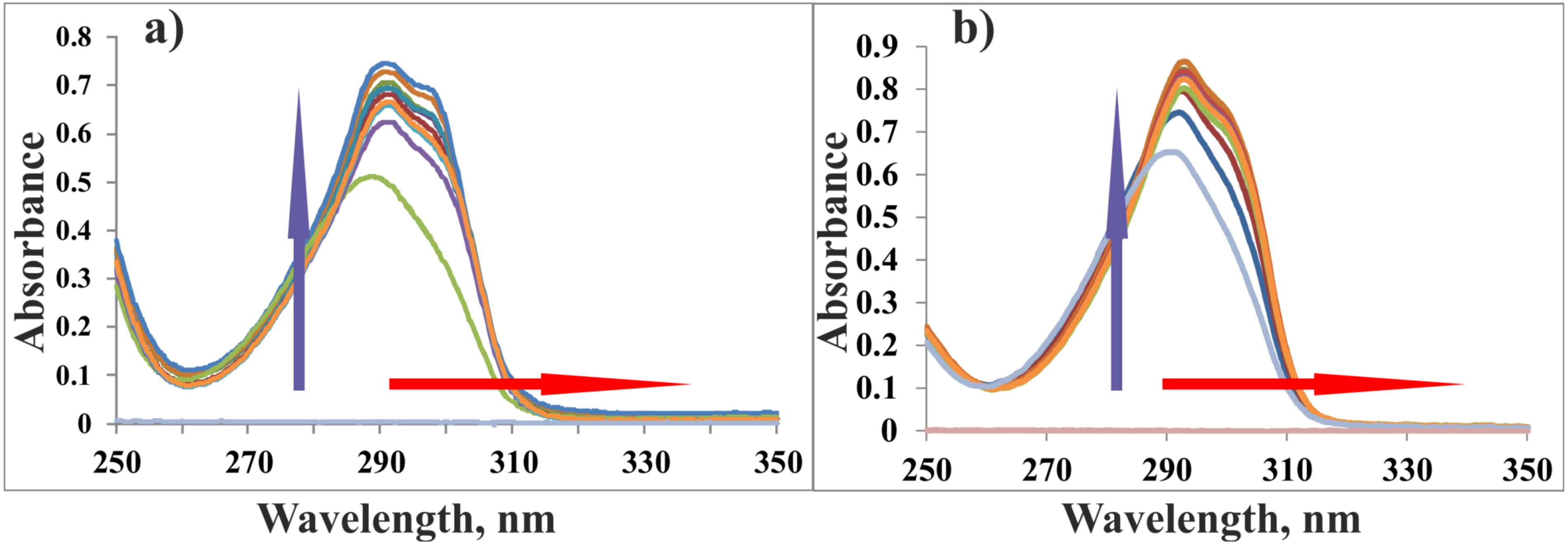

Fig. S4. Bindfit (Fit data to 1:1, 1:2 and 2:1 Host-Guest equilibria) Screenshots taken from the summary window of the website supramolecular.org. This screenshots shows the raw data for UV-vis titration of 1 with SDS, the data fitted to 1:1 binding model (a), 1:2 binding model (b) and 2:1 binding model (c).

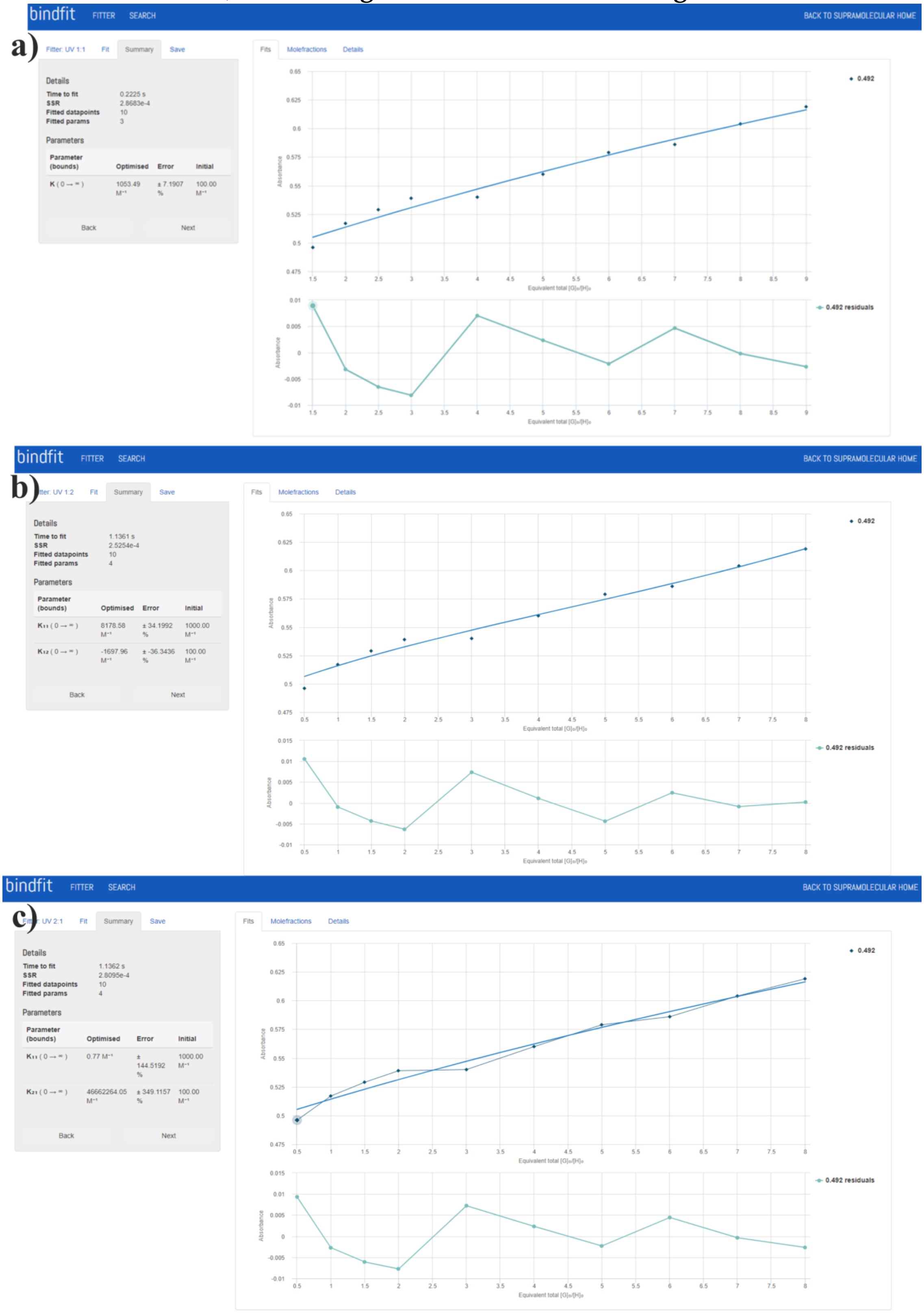

Fig. S5. Bindfit (Fit data to 1:1, 1:2 and 2:1 Host-Guest equilibria) Screenshots taken from the summary window of the website supramolecular.org. This screenshots shows the raw data for UV-vis titration of 2 with DTAC, the data fitted to 1:1 binding model (a), 1:2 binding model (b) and 2:1 binding model (c).

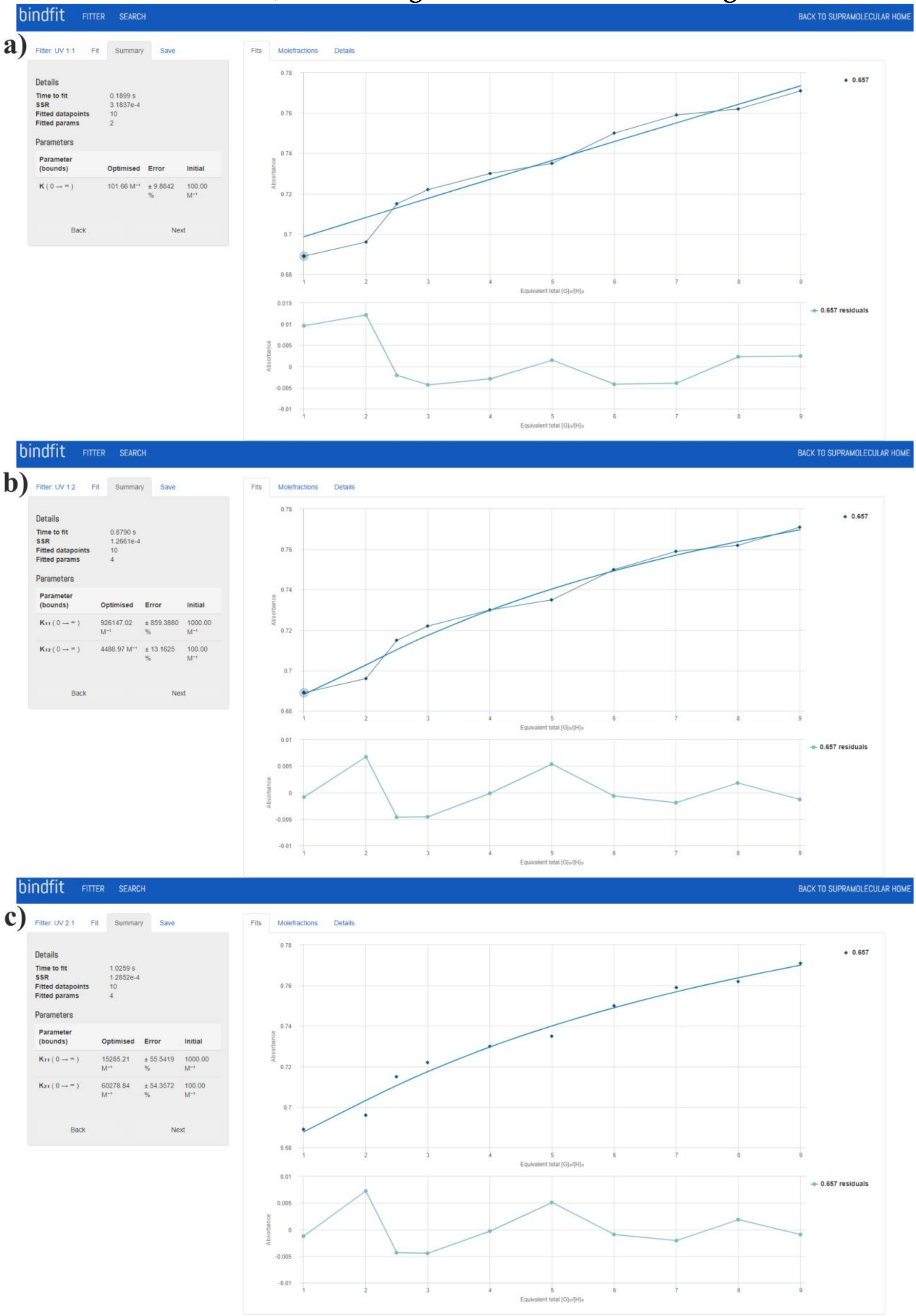

Job Plots

Series of the solutions of pillar[5]arene derivatives **1** and **2** and guests (SDS and DTAC) were prepared in water. The volume ratio of the host and guest solutions varied from 0.6:2.4 to 2.4:0.6, respectively, with the total concentration of the host (H) and guest (G) being constant and equal to $3{\cdot}10^{-5}$ M. The solutions were used without further stirring. The absorbance Ai of the complexation systems was measured at the maximum absorbance wavelength of the complex. The absorbance values were used to plot a diagram from which maximum the structures of the complexes were deduced. Three independent experiments were carried out for each system.

Fig. S6. a) the Job's plot for the determination of the stoichiometry in the complex of the system pillar[5]arene 1 and SDS in water; b) the Job's plot for the determination of the stoichiometry in the complex of the system pillar[5]arene 2 and DTAC in water.

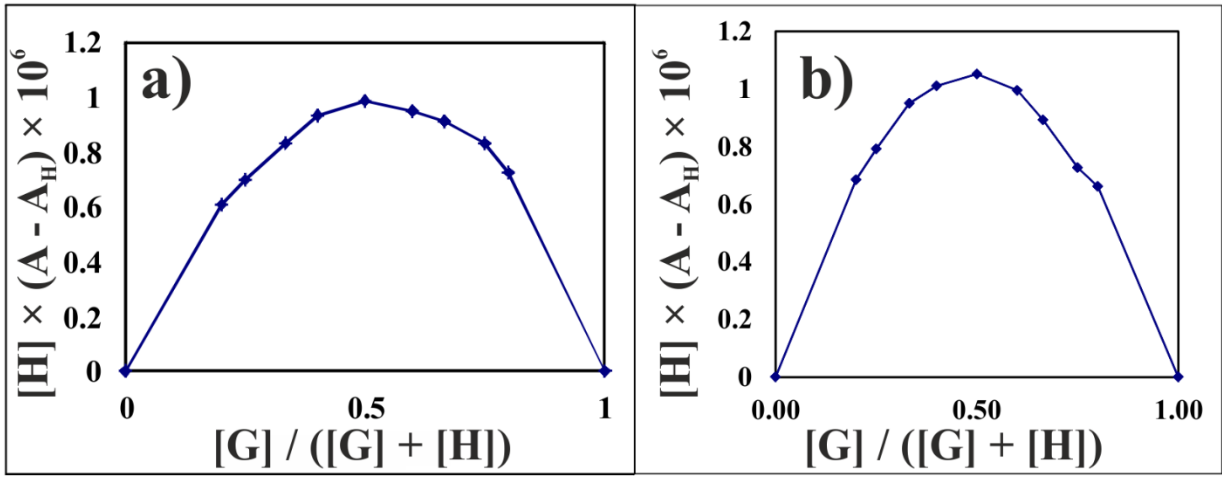

Table S2. Sizes of aggregates formed from compound 1 and sodium dodecyl sulfate (SDS), their corresponding polydispersity indices (PDI) and ζ-potential.

C_{pillar}, M		Pillar[5]arene-surfactant ratio			
		1:1	1:10	1:100	1:1000
1×10^{-6}	d, nm	183	506	1270	71
	PDI	0.42±0.11	0.45±0.11	0.82±0.18	0.16±0.04
	ζ, mV	-[b)]	-[b)]	-[b)]	-40.2
1×10^{-5}	d, nm	125	783	242	338
	PDI	0.34±0.08	0.95±0.05	0.13±0.01	0.61±0.17
	ζ, mV	-[b)]	-[b)]	-20.5	-[b)]
1×10^{-4}	d, nm	190	-[a)]	-[a)]	329
	PDI	0.34±0.08			0.60±0.21
	ζ, mV	-[b)]			-[b)]
1×10^{-3}	d, nm	224	-[a)]	-[a)]	453
	PDI	0.34±0.07			0.65±0.24
	ζ, mV	41.3			-[b)]

-[a)] precipitation falls

-[b)] did not determine the value of ζ-potential

Table S3. Sizes of aggregates formed from compound 2 and dodecyltrimethylammonium chloride (DTAC), their corresponding polydispersity indices (PDI) and ζ-potential.

C_{pillar}		Pillar[5]arene-surfactant ratio			
		1:1	1:10	1:100	1:1000
1×10^{-6}	d, nm	354	3060	256	233
	PDI	0.42±0.05	0.87±0.15	0.46±0.18	0.37±0.02
	ζ, mV	-[b)]	-[b)]	-[b)]	-[b)]
1×10^{-5}	d, nm	314	254	584	170
	PDI	0.42±0.08	0.83±0.04	0.50±0.30	0.27±0.05
	ζ, mV	-[b)]	-[b)]	-[b)]	2.54
1×10^{-4}	d, nm	178	286	245	332
	PDI	0.31±0.03	0.23±0.01	0.77±0.09	0.52±0.13
	ζ, mV	-39.4	-34.7	-[b)]	-[b)]
1×10^{-3}	d, nm	235	374	942	437
	PDI	0.37±0.02	0.71±0.13	0.78±0.16	0.49±0.23
	ζ, mV	-30.7	-[b)]	-[b)]	-[b)]

-[a)] precipitation falls

-[b)] did not determine the value of ζ-potential

Fig. S7. Electron absorption spectra of pillar[5]arene 1/DAPI in equimolar ratio in water ($c_{macrocycle}$ = 3×10^{-5} M, c_{guest} = 3×10^{-5} M).

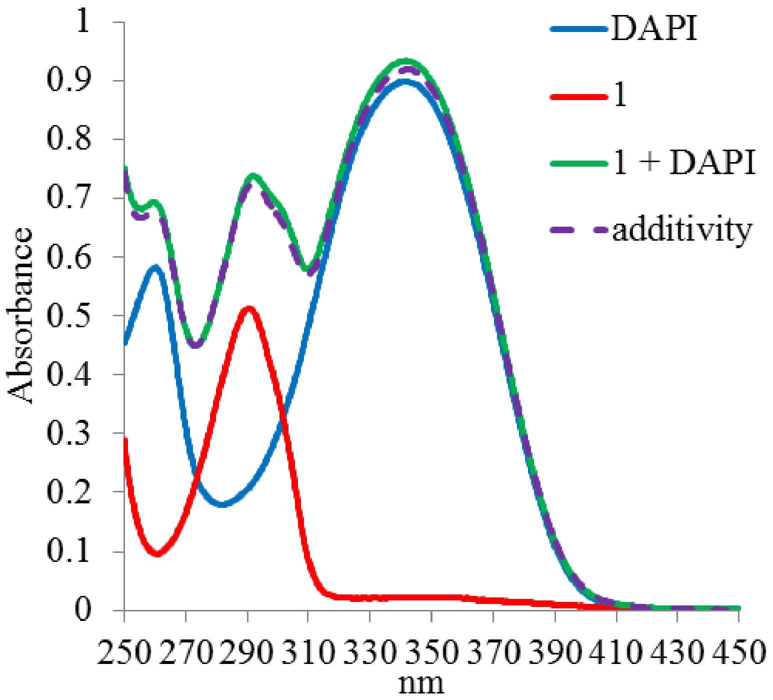

Table S4. Sizes of aggregates of interpolyelectrolyte complexes (IPEC-1 and IPEC-2) formed by macrocycles 1 and 2 with respect to DAPI, their corresponding polydispersity indices (PDI) and ζ-potential.

	d, nm	PDI	ζ, mV
SIPEC-2/DAPI	494	0.73±0.17	-[b)]
NIPEC-2/DAPI	683	0.27±0.03	24
SIPEC-1/DAPI	216	0.06±0.03	-36.3
NIPEC-1/DAPI	215	0.55±0.09	-[b)]

-[b)] did not determine the value of ζ-potential

Fig. S8. Size distribution of self-assembles of: a) SIPEC-1/DAPI in water; b) NIPEC-2/DAPI in water.

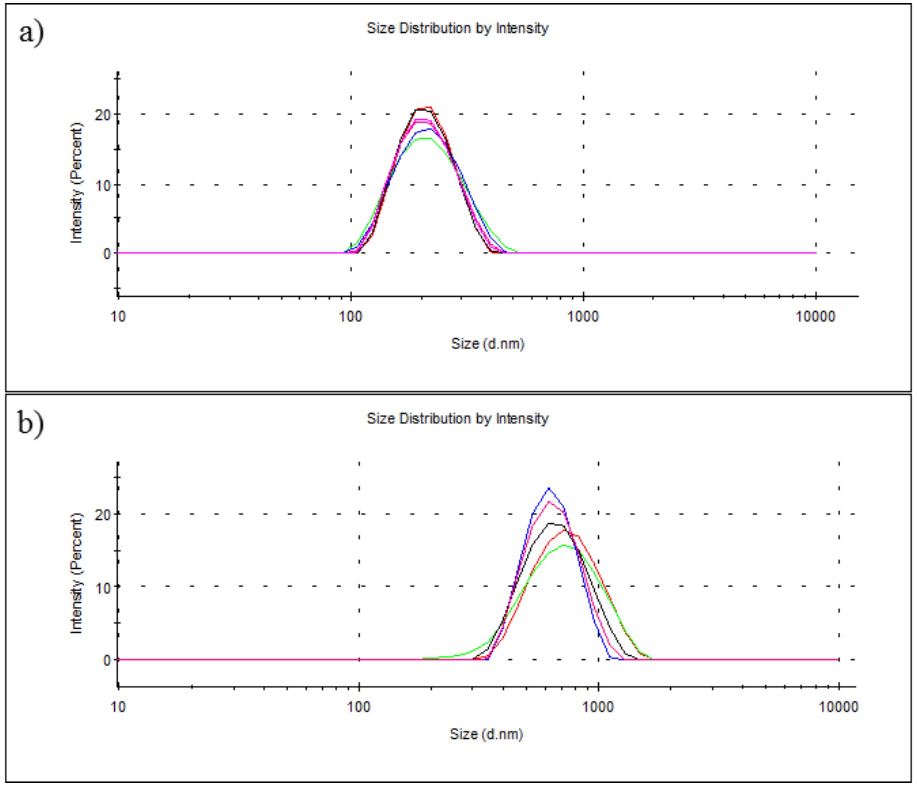

Fig. S9. Luminescence spectra: a) SDS (3×10^{-2} M) + DAPI (3×10^{-5} M); b) NIPEC-1 + DAPI (3×10^{-5} M).

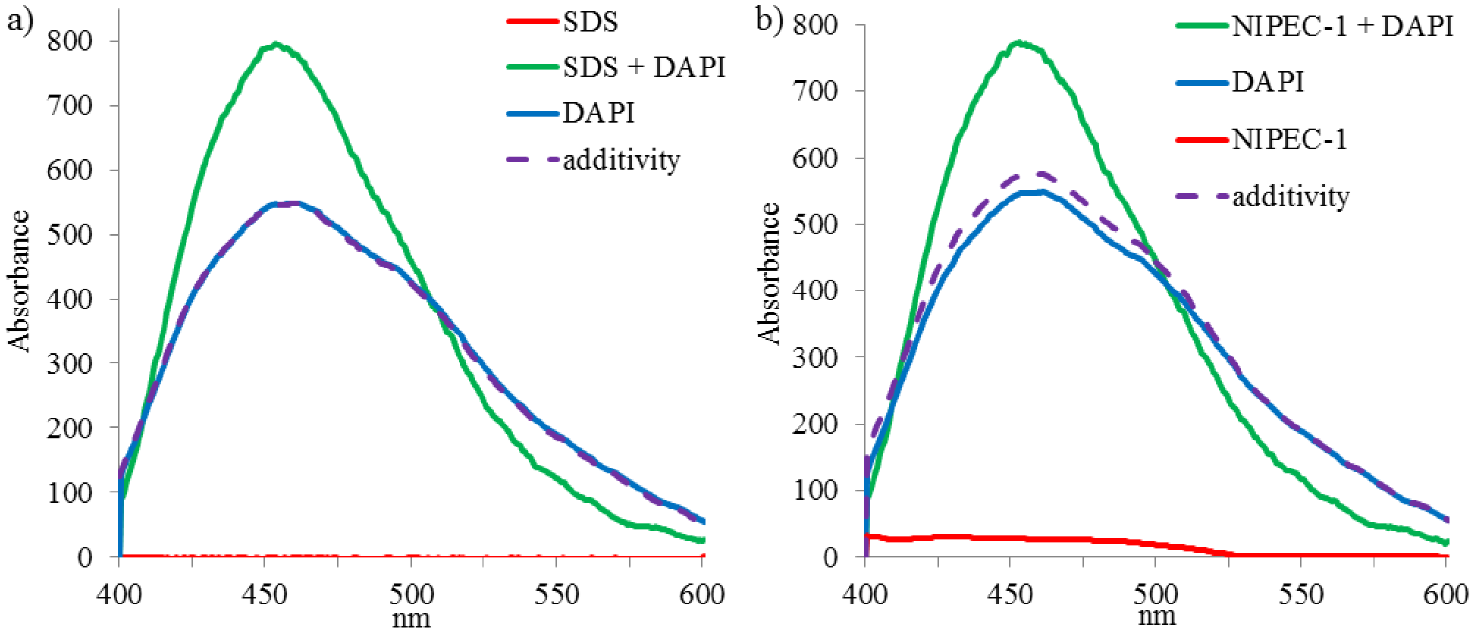

Fig. S10. Luminescence spectra: a) SIPEC-2 + DAPI (3×10^{-5} M); b) DTAC (3×10^{-3} M) + DAPI (3×10^{-5} M); c) DTAC (3×10^{-2} M) + DAPI (3×10^{-5} M); d) NIPEC-2 + DAPI (3×10^{-5} M).

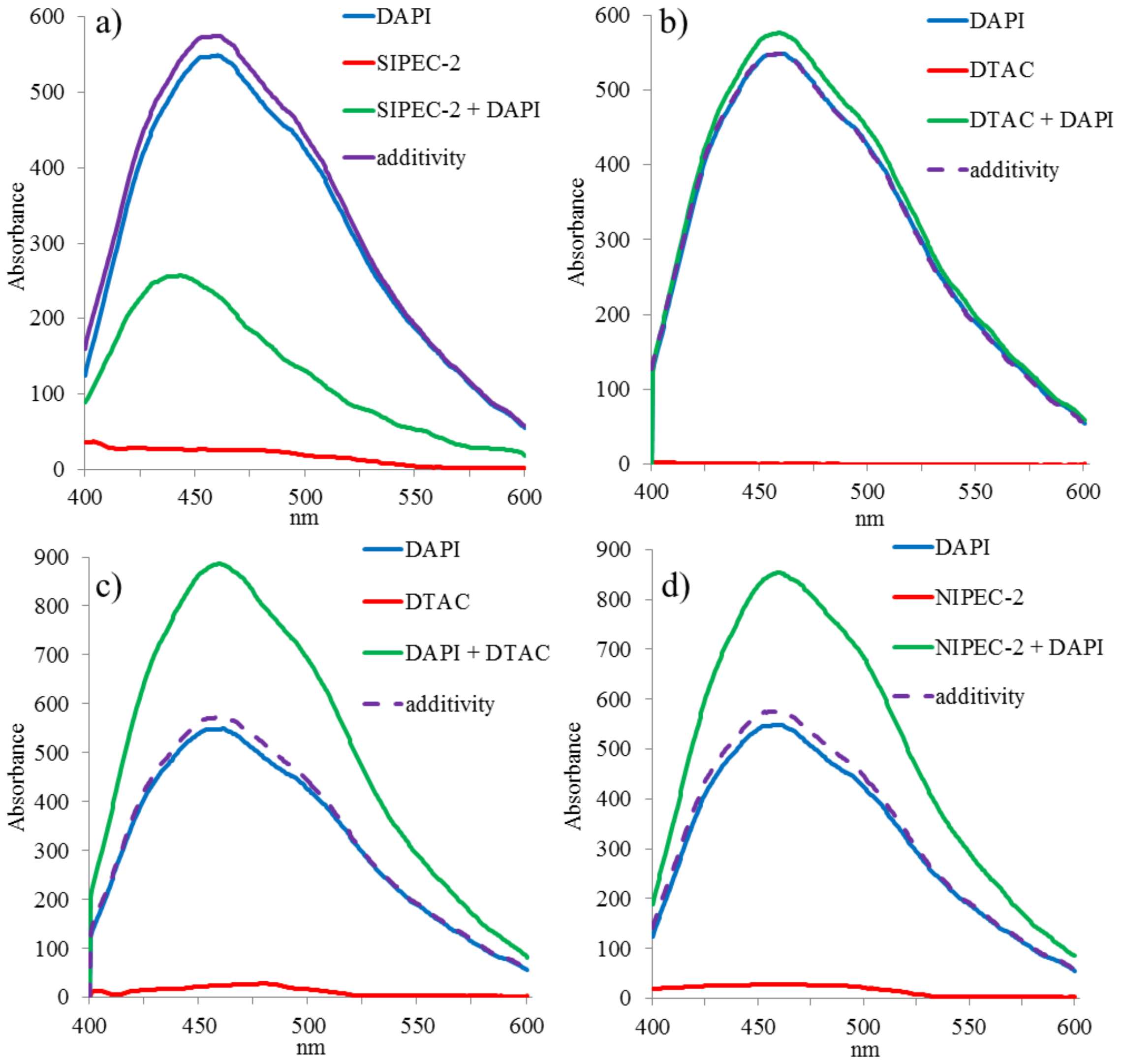

Table S5. Dependence of the size (D_h) and particles concentration in the 2/DAPI system at various extinguishing filters on their molar ratio.

Molar ratio DAPI/2	No emission filter		430 nm emission filter		500 nm emission filter	
	D_h (nm)	Number of particle 1×10[8]	D_h (nm)	Number of particle 1×10[8]	D_h (nm)	Number of particle 1×10[8]
1	173±5	12.1±0.5	244±157	1.41±0.2	203±30	1.99±0.2
1.5	140±4	19.4±0.3	294±45	1.27±0.3	297±18	1.57±0.2
1.75	90±2	22.8±0.5	187±40	1.24±0.4	230±26	1.18±0.3
2	94±8	25.7±3.43	305±62	0.57±0.2	250±11	0.88±0.1
3	**86**±3	31.1±0.4	**88**±22	2.12±0.2	**83**±8	3.05±1.8

Fig. S11. Size and concentration of nanoparticles determined in solution by the NTA method in the DAPI/2 system at different molar ratios.

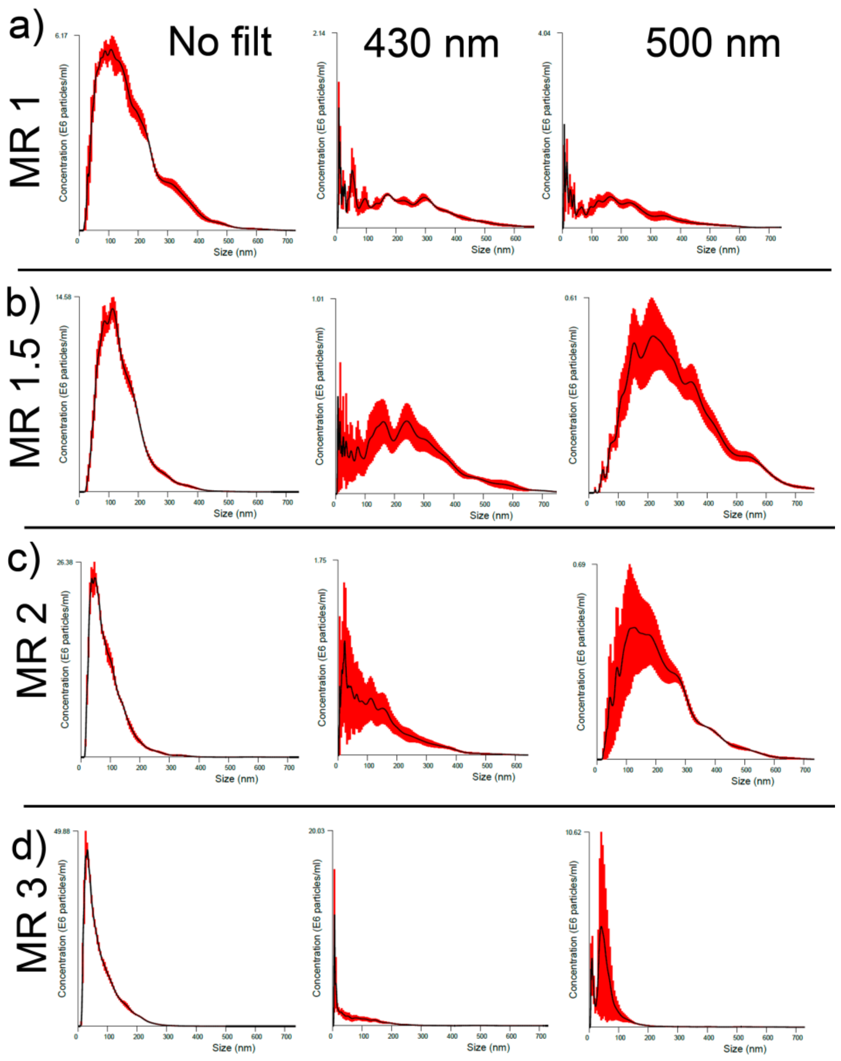

Fig. S12. View of the energy geometry optimized complex formed by macrocycle **2** and 4′,6-diamidino-2-phenylindole (DAPI) calculated by DINC 2.0 web server: a) the dye is inside the cavity; b), c) the dye is outside the cavity.

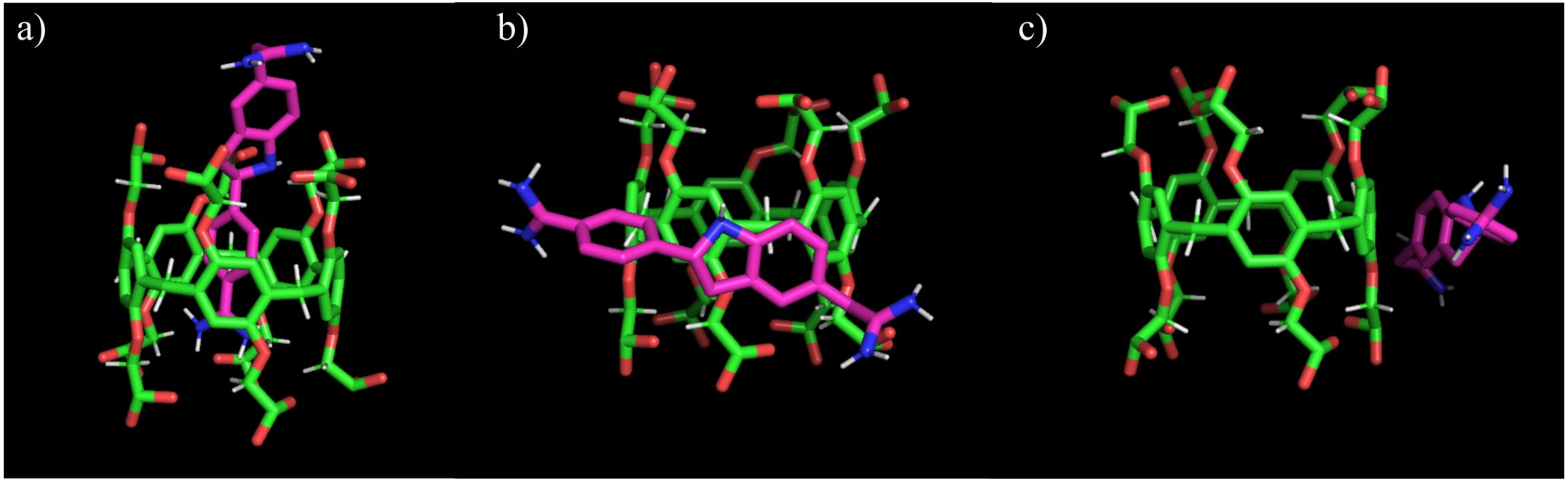

Supplement: Supplementary file 1 [file ijms-22-06038-s001.zip › ijms-1246856-supplementary.pdf]
